# Objective Risk in Trust Decisions Under Varying Social Distance: An Exploratory fMRI Study

**DOI:** 10.3390/bs16050695

**Published:** 2026-05-02

**Authors:** Ying Chen, Chengru Zhao, Xia Wu

**Affiliations:** 1 Department of Psychology, Tianjin University of Technology and Education, Tianjin 300222, China; 2 Faculty of Psychology, Tianjin Normal University, Tianjin 300387, China

**Keywords:** trust decision-making, objective risk, social distance, neural correlates, trust game, exploratory fMRI study

## Abstract

Trust decisions involve both social evaluation and uncertainty processing, yet standard trust game paradigms do not fully dissociate trust-specific social computation from more general risk- and value-related processes. In this exploratory whole-brain fMRI study, we examined how objective risk and social distance were associated with trust decisions within a 2 × 2 trust game. Twenty-three adults completed the task, and 20 were included in the fMRI analyses after exclusion for excessive head motion. Behaviorally, trust rates were significantly lower under high than low objective risk, whereas neither the main effect of social distance nor the interaction between objective risk and social distance was significant. Relative to baseline, task performance engaged prefrontal and parietal regions. Compared with distrust decisions, trust decisions were associated with greater activation in prefrontal and visual regions, along with stronger negative activation in the insula. Objective risk was associated with differential activation in temporal, supramarginal, and precentral regions. Under the present manipulation, we did not observe significant neural modulations by social distance. These findings suggest that, in this low-context paradigm, objective risk was a more robust source of behavioral and neural variation than social distance. Given the exploratory design, modest sample size, and the task’s limited ability to separate social trust from generic risk/value processing, the findings should be interpreted cautiously.

## 1. Introduction

Trust is a socially embedded decision to accept vulnerability under conditions of incomplete information and the possibility of exploitation, based on expectations about another person’s future conduct (Mayer et al., 1995; Rousseau et al., 1998). In contemporary social and organizational settings, including digital platforms and human–AI interaction, trust is often treated as a precondition for cooperation, coordination, and the accumulation of social capital (Fukuyama, 1996; Uslaner, 2002). At the same time, trust is highly context-sensitive: it is shaped not only by institutional and relational cues but also by how uncertainty and potential losses are represented within the decision environment (Chaudhary et al., 2021; H. Hu, 2007; Lin et al., 2016; Pan & Houser, 2019). Understanding how contextual factors shape trust is therefore important both for psychological theories of social decision-making and for applied settings in which people must decide whether to rely on others under uncertainty.

One factor that may shape trust is objective risk. Objective risk refers to an externally specified probability structure over gains and losses, rather than a purely subjective feeling of uncertainty (Knight, 1921; Slovic, 1987). In trust games and related decision settings, higher objective risk is often associated with lower willingness to trust (Bohnet & Zeckhauser, 2004; Yang et al., 2015). At the same time, an important conceptual limitation of standard trust game paradigms is that they do not fully dissociate trust-specific social computation from more general expected-value or risk-related processing. In other words, what appears behaviorally as “less trust” may partly reflect generic aversion to probabilistic loss or changes in expected value, rather than a purely social judgment about another person’s trustworthiness (Chetty et al., 2020). Accordingly, the present study does not seek to claim a process-pure isolation of social trust. Instead, we examine trust decisions under different objective risk contexts while acknowledging that such decisions may reflect blended processing of social beliefs and nonsocial risk/value computations.

At the neural level, prior work suggests that trust under uncertainty recruits distributed systems involved in valuation, cognitive control, and social inference, including prefrontal and parietal–temporal regions (Miller & Cohen, 2001; Bressler & Menon, 2010; Chen et al., 2020; Krueger & Meyer-Lindenberg, 2019). In particular, prefrontal regions have been linked to goal-directed evaluation and control, whereas parietal–temporal regions may contribute to integrating social and probabilistic information during interpersonal choice (Miller & Cohen, 2001; Bressler & Menon, 2010; Chen et al., 2020). Electrophysiological evidence further suggests that changes in risk context during trust decisions are accompanied by altered prefrontal and temporoparietal activity (X. Hu et al., 2018). However, it remains unclear whether objective risk primarily alters expected-value computation, heightens vigilance toward a partner’s intentions, or changes how the interaction is evaluated more broadly (Krueger & Meyer-Lindenberg, 2019). These considerations motivate work examining objective risk as an important contextual determinant of trust, while avoiding overly strong claims about process specificity.

A second factor that may shape trust is social distance. Social distance refers to perceived psychological closeness between interaction partners and may be cued by shared identity, group affiliation, or relational history. In experimental work, trust is often higher toward socially closer or in-group partners (Binzel & Fehr, 2013; Rosenberger et al., 2020). Related research also suggests that distance cues can influence vigilance and social judgment in broader socio-technical settings (Martin et al., 2021). Neural accounts have often associated social-distance-related processing with parietal–temporal and medial frontal systems involved in representing other people and evaluating social relationships (Bressler & Menon, 2010; Chen et al., 2020). Nevertheless, the effects of social distance depend strongly on how distance is instantiated. They are likely to be more robust when supported by richer identity cues, repeated interaction, or stronger relational relevance, and may be attenuated in relatively low-context, single-shot settings (Binzel & Fehr, 2013; Rosenberger et al., 2020; Pan & Houser, 2019). In the present study, social distance is therefore treated as a theoretically relevant but potentially subtle influence.

A further question is whether objective risk and social distance operate independently or jointly during trust decisions. Some perspectives suggest interaction: betrayal aversion implies that the subjective weight of potential loss may depend on relational context, and construal-level accounts suggest that greater distance may shift attention toward an abstract probabilistic structure (Bohnet & Zeckhauser, 2004; Loewenstein et al., 2001; Trope & Liberman, 2010). Likewise, findings on in-group favoritism suggest that relational proximity can sometimes buffer sensitivity to uncertainty in cooperative settings (Balliet et al., 2014). At the same time, value-integration and large-scale systems accounts leave open the possibility that social and nonsocial inputs may contribute in partly additive, rather than strongly interactive, ways (Levy & Glimcher, 2012; Bressler & Menon, 2010; Chen et al., 2020). Empirically, this question remains unsettled, particularly in paradigms that manipulate objective risk and social distance within the same task.

Against this background, the present study used a 2 × 2 trust game design to examine how objective risk (low vs. high) and social distance (near vs. far) were associated with trust decisions within a single exploratory fMRI paradigm. Objective risk was defined in advance by die-roll success probability and implemented in the task through return-stage outcomes following trust choices, whereas social distance was cued by partner affiliation. Behaviorally, we tested whether trust rates varied as a function of risk and distance. Neurally, we used whole-brain analyses to examine task-related activation, decision-related activation, and condition-related differences associated with trust, objective risk, and social distance. Based on the prior literature, we expected a higher objective risk to be associated with lower trust rates. We also considered the possibility that social closeness might be associated with higher trust, although any such effect could be limited under the present low-context manipulation. At the neural level, we expected trust decisions under varying task contexts to engage prefrontal regions involved in evaluation and control, as well as parietal–temporal regions involved in integrating social and probabilistic information; we also considered salience-related regions such as the insula as relevant to distrust or heightened uncertainty. Given the exploratory design, modest sample size, and the paradigm’s limited ability to separate social trust from generic risk/value processing, our goal was not to draw strong mechanistic conclusions, but rather to characterize how these two contextual factors were associated with behavioral and neural variation in trust decisions under the present task parameters.

## 2. Methods

### 2.1. Participants

We report how we determined our sample size, all data exclusions, all manipulations, and all measures in this study.

Twenty-three right-handed undergraduates (5 men; age range = 19–25 years; M = 21.2 years, SD = 1.3) from a local university participated. An a priori power analysis conducted in G*Power 3.1 for a 2 × 2 within-subjects ANOVA (*α* = 0.05, assumed correlation among repeated measures *r* = 0.50, nonsphericity correction *ε* = 1.00) indicated that a medium effect size (*f* = 0.25) would require N = 24 to achieve 1 − *β* = 0.80. Our enrolled sample of 23 approached this target. All participants had normal or corrected-to-normal vision and reported no history of neurological or psychiatric disorders. This study was approved by the Institutional Review Board of our university (Approval No. 2025030325) and was conducted in accordance with the Declaration of Helsinki. Written informed consent was obtained from all participants prior to participation. Compensation consisted of a fixed show-up payment plus a performance-contingent bonus drawn from randomly selected trials.

Three participants were excluded from the fMRI analyses because of excessive head motion, leaving a final neuroimaging sample of N = 20. Unless otherwise noted, behavioral analyses refer to the full sample (N = 23).

### 2.2. Trust Game Paradigm

Before the formal fMRI task, participants completed a practice session to become familiar with the task procedure, rules, and response keys. Participants were instructed that the formal task followed the same rules as the practice task and that they would interact with partners from two groups, one from their own university and one from another university.

The task used a 2 × 2 factorial design crossing Social Distance and Objective Risk. Social Distance included two levels: near social distance, in which the partner was described as being from the participant’s own university, and far social distance, in which the partner was described as being from another university. Objective Risk included two levels: high risk and low risk. Thus, the task included four conditions: near social distance/high risk, near social distance/low risk, far social distance/high risk, and far social distance/low risk.

**Figure 1:**
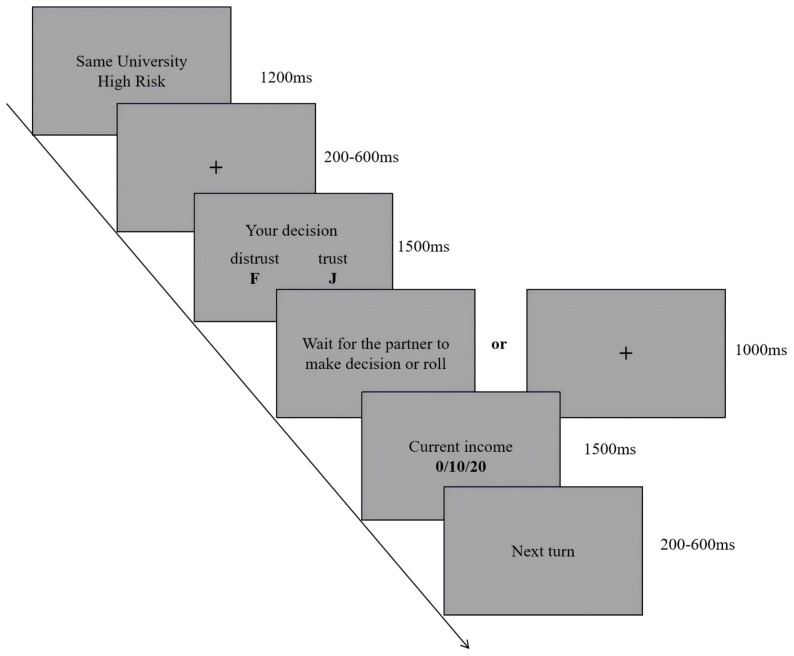
Trust game trial structure and factorial manipulations. Social distance was cued by partner affiliation (Near: own university; Far: different university). Objective risk was defined in the task instructions by die-roll success probability (Low risk = 5/6; High risk = 1/2). Participants then chose Trust or Not Trust. After a Trust choice, the trial entered a return stage in which the participant received either 0 or 20. A return of 20 indicated that the trustee chose to split the payoff and that the die roll was successful, whereas a return of 0 could result either from trustee betrayal or from an unsuccessful die roll. Therefore, the realized return proportions did not exactly match the die-roll probabilities. For example, under High risk, returns of 0 and 20 occurred at a ratio of 48:32 (3:2), making zero-return outcomes more frequent in the realized feedback. After Not Trust, the participant retained the default payoff.

Each trial (Figure 1) began with a cue indicating both the social-distance condition and the objective-risk condition for the current investment decision. The cue was presented for 1200 ms, followed by a central fixation cross presented for 200–600 ms. Participants then made a trust decision on a response screen displaying the options “Trust” and “Not Trust”. The decision screen was presented for 1500 ms. The response keys were counterbalanced across participants: for half of the participants, the F key corresponded to Trust and the J key corresponded to Not Trust, whereas for the other half, this assignment was reversed.

After the participant made a choice, a 1000 ms waiting screen was presented. If the participant chose Trust, the screen indicated that the partner was making a decision and that the participant should wait for the partner’s decision or the die-roll outcome. If the participant chose Not Trust, a central fixation cross was presented for the same duration. Feedback was then presented for 1500 ms. Finally, a next trial prompt was presented for 200–600 ms, after which the next trial began. The full task consisted of 160 trials, with 40 trials in each cell of the 2 × 2 design.

**Objective risk manipulation.** Objective risk was defined in the task instructions by the die-roll success probability. In the low-risk condition, the stated probability of a successful die roll was 5/6, whereas in the high-risk condition, the stated probability was 1/2. If participants chose Not Trust, they always received a fixed payoff of 10 points for that trial. If participants chose Trust, they received either 0 or 20 points. Participants were instructed that a 20-point outcome indicated a successful return, whereas a 0-point outcome could result from either trustee non-reciprocation or an unsuccessful die roll.

The realized feedback outcomes were implemented according to a preset return schedule in the experimental program. Within each social-distance condition, high-risk trials included 24 zero-return outcomes and 16 positive-return outcomes, whereas low-risk trials included 10 zero-return outcomes and 30 positive-return outcomes. Thus, across the 80 high-risk trials, Trust choices were followed by 0-point and 20-point outcomes according to a preset ratio of 48:32; across the 80 low-risk trials, the corresponding ratio was 20:60.

The preset return schedule was used to operationalize objective risk in the fMRI task. No independent trustee reciprocation probabilities were modeled separately; instead, trustee-related and die-roll-related outcomes were represented jointly in the preset feedback schedule.

**Social distance manipulation.** Social distance was cued by university affiliation. “Near” partners were described as students from the participant’s own university, whereas “Far” partners were described as students from a different university. Although the two universities were located in the same city, we used affiliation as a cue to psychological rather than geographic distance. Partner cues included an affiliation badge and anonymized initials, and partner photographs were matched on age and gender. Participants were told that partner identities were anonymized and drawn from prior sessions.

To support the plausibility of this manipulation, we conducted a separate anonymized online pilot survey before the fMRI study with 37 undergraduates (9 men; age range = 20–25 years; M = 21.4 years, SD = 0.8). Participants rated perceived psychological distance (1 = very close, 10 = very distant) toward two target groups: students from their own university and students from other universities. Ratings indicated lower perceived distance for own-university students (M = 3.62, SD = 2.41) than for other-university students (M = 7.08, SD = 2.13), *t*(36) = −5.944, *p* < 0.001, *d* = 0.977. This pilot was conducted in a separate sample and was used only to support the plausibility of the affiliation-based cue; no within-sample manipulation check was obtained during scanning.

### 2.3. Procedure

This study comprised a behavioral training session followed by an fMRI session on the same day. The trust game task was programmed in E-Prime 3.0 (Psychology Software Tools, Pittsburgh, PA, USA). After consent and screening, participants completed a practice block with feedback and a comprehension quiz covering payoff contingencies, objective-risk probabilities, and the meaning of the affiliation cues. During desktop practice, response mapping was counterbalanced across participants.

Before scanning, participants completed a brief refresher practice block (20 trials). During scanning, stimuli were back-projected onto a screen at the rear of the bore (60 Hz; 1024 × 768 resolution) and viewed through a mirror mounted on the head coil. MRI-compatible corrective lenses were provided when necessary. Responses were collected with an MRI-compatible two-button fiber-optic response device, and response mapping was counterbalanced across participants. Foam padding was used to minimize head motion, and ear protection was provided.

### 2.4. fMRI Data Acquisition

MRI data were acquired on a Siemens Prisma 3T scanner (Siemens, Erlangen, Germany) equipped with a 64-channel head coil. All images were acquired in the axial plane parallel to the anterior commissure–posterior commissure (AC–PC) line. Head motion was minimized with foam padding.

For each participant, three runs of functional images were collected using a T2*-weighted gradient-echo echo-planar imaging (EPI) sequence sensitive to blood oxygenation level-dependent (BOLD) contrast. Each run contained 522 stored volumes with the following parameters: repetition time (TR) = 2000 ms, echo time (TE) = 30 ms, flip angle = 90°, field of view (FOV) = 224 × 224 mm^2^, matrix = 64 × 64, voxel size = 2 × 2 × 2 mm^3^, and 62 axial slices with no gap. In addition, two initial dummy scans were acquired by the scanner at the beginning of each run for T1 equilibration. During preprocessing, the first 10 stored volumes of each run were discarded to allow magnetization and task-related signal stabilization before statistical analysis.

A high-resolution structural image was acquired for each participant using a T1-weighted magnetization-prepared rapid gradient-echo (MPRAGE) sequence with the following parameters: TR = 2530 ms, TE = 2.98 ms, flip angle = 7°, FOV = 224 × 256 mm^2^, matrix = 256 × 256, voxel size = 0.9 × 0.9 × 0.9 mm^3^, and 192 axial slices with no gap.

### 2.5. Behavioral Data Analysis

Trials with missed responses or implausible reaction times (<200 ms or >3 SD above a participant’s mean) were excluded from behavioral summaries. For each participant, the trust rate was computed as the proportion of Trust choices in each cell of the 2 × 2 design.

Behavioral effects were evaluated using a 2 × 2 repeated-measures analysis of variance (ANOVA) with Objective Risk (High vs. Low) and Social Distance (Near vs. Far) as within-subject factors and trust rate as the dependent variable. Because each factor had only two levels, sphericity was not an issue. We report *F* values, *p* values, and partial eta-squared (ηp^2^).

### 2.6. fMRI Data Analysis

***Preprocessing.*** Neuroimaging data were preprocessed in SPM12 (Wellcome Trust Centre for Neuroimaging, London, UK; RRID: SCR_007037). When necessary, T1-weighted and EPI images were manually reoriented to the AC–PC plane. For each participant, preprocessing included (1) discarding the first 10 stored functional volumes of each run; (2) slice-timing correction; (3) realignment of all EPI volumes to the first retained volume to estimate six rigid-body motion parameters; (4) co-registration of the mean EPI image to the participant’s T1 anatomical image using normalized mutual information; (5) segmentation of the T1 image into gray matter, white matter, and cerebrospinal fluid; (6) nuisance regression including white-matter and cerebrospinal-fluid signals together with the 24-parameter head-motion model; (7) normalization to MNI space using the deformation fields from segmentation, resampled to 3 × 3 × 3 mm^3^; and (8) spatial smoothing with an 8 mm full-width at half-maximum Gaussian kernel.

Participants with excessive head motion (>3° rotation) were excluded from group analyses.

***General linear modeling (GLM)*.** First-level statistical analyses were conducted using the general linear model (GLM). Decision events were modeled at the onset of the choice screen and were sorted into eight regressors defined by Choice (Trust vs. Not Trust), Objective Risk (High vs. Low), and Social Distance (Near vs. Far): Trust–High–Near, Trust–Low–Near, Trust–High–Far, Trust–Low–Far, Not Trust–High–Near, Not Trust–Low–Near, Not Trust–High–Far, and Not Trust–Low–Far. In addition, the condition cue and outcome feedback were modeled as separate task regressors. The six realignment parameters were entered as nuisance covariates. All regressors were convolved with the canonical hemodynamic response function (HRF), and a high-pass filter of 1/128 Hz was applied.

At the group level, participant-specific contrast maps were entered into random-effects analyses. The primary whole-brain contrasts included task > baseline, decision > baseline, trust > distrust, high risk > low risk, and near > far. We also examined the reverse social-distance contrast, far > near, and the Risk × Social Distance interaction contrasts in both directions. Given the exploratory nature of the study, whole-brain statistical inference used AlphaSim cluster-extent correction. With a voxel-wise threshold of *p* < 0.01, Monte Carlo simulation indicated that clusters of at least 322 contiguous voxels were required to achieve a corrected significance level of *p* < 0.05.

## 3. Results

### 3.1. Behavioral Results

**Figure 2:**
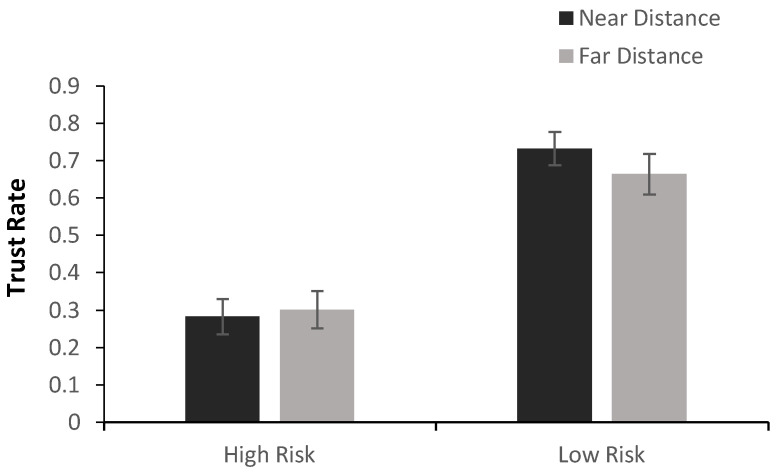
The impact of objective risk and social distance on trust rate.

A 2 × 2 repeated-measures ANOVA was conducted on the trust rate with Objective Risk (High vs. Low) and Social Distance (Near vs. Far) as within-subject factors (Figure 2). The analysis revealed a significant main effect of objective risk, *F*(1,22) = 49.709, *p* < 0.001, ηp^2^ = 0.693. Across social-distance conditions, trust rates were lower under high objective risk (*M* = 0.29) than under low objective risk (*M* = 0.70). By contrast, the main effect of social distance was not significant, *F*(1,22) = 0.585, *p* = 0.450, nor was the Objective Risk × Social Distance interaction, *F*(1,22) = 1.927, *p* = 0.179. As shown in Figure 2, the reduction in trust under high-risk conditions was apparent for both near and far partners.

### 3.2. fMRI Results

Three participants were excluded because of excessive head motion; thus, all fMRI analyses were conducted on the remaining 20 participants.

#### 3.2.1. Task-Related Activation (Task > Baseline)

**Figure 3:**
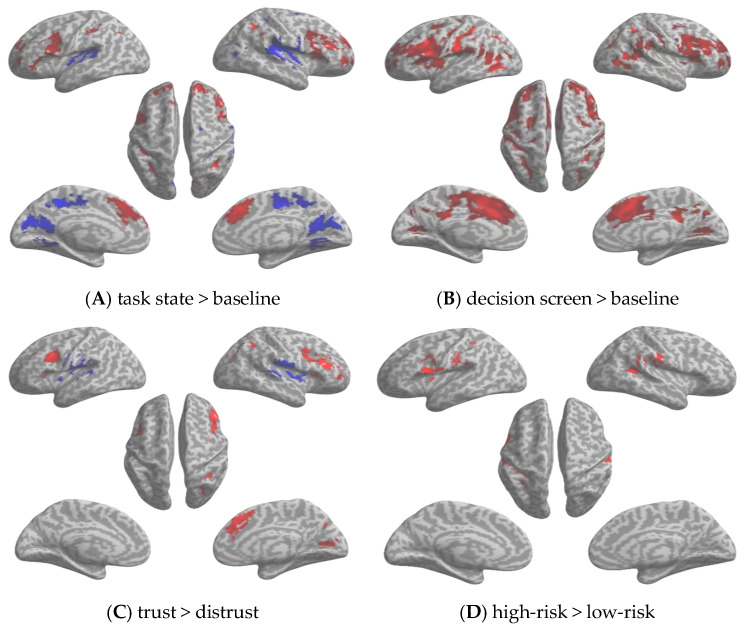
Brain region activation in the trust game task. (**A**) Whole-brain activation during the trust game (task > baseline). (**B**) Decision epoch activity (decision screen > baseline). (**C**) Trust versus distrust decisions. (**D**) Main effect of objective risk (high > low). Red indicates positive activation; blue indicates negative activation.

We first examined whole-brain activation during the trust game task relative to baseline (Figure 3A, Table 1). Positive clusters were observed in the angular gyrus (AG), supramarginal gyrus (SMG), superior frontal gyrus (SFG), inferior frontal gyrus (IFG), middle frontal gyrus (MFG), middle occipital gyrus (MOG), inferior parietal lobule (IPL), and precentral gyrus. Negative clusters were observed in the insular cortex, superior temporal gyrus (STG), postcentral gyrus, and precuneus. Accordingly, this contrast is described here in terms of the specific frontal, parietal, temporal, occipital, insular, and precuneus clusters listed in Table 1.

#### 3.2.2. Decision-Related Activation (Decision Screen > Baseline)

We next isolated activity associated with the decision phase (Figure 3B, Table 2). This contrast showed large positive clusters in the precentral gyrus and IPL, together with additional activation in the MFG, superior frontal gyrus, orbital part, middle frontal gyrus, orbital part, and the cerebellar vermis. Thus, decision-related activation was primarily observed in frontal, parietal, and cerebellar regions.

#### 3.2.3. Trust-Versus-Distrust Decisions

To characterize choice-related differences, we directly compared trust and distrust decisions (Figure 3C, Table 3). Relative to distrust, trust decisions were associated with greater activation in the IFG, MFG, medial superior frontal gyrus, cingulate gyrus, superior occipital gyrus (SOG), fusiform gyrus, calcarine sulcus, lingual gyrus, and precentral gyrus. Negative clusters in the trust > distrust contrast were observed in the STG, insula, and SMG. These negative clusters indicate relatively greater activation for distrust than for trust.

#### 3.2.4. Effects of Objective Risk and Social Distance

We then examined condition-related modulation by the two task factors. For objective risk (high > low), significant positive clusters were observed in the middle temporal gyrus (MTG), SMG, STG, and precentral gyrus (Figure 3D, Table 4). Thus, under the present task parameters, objective risk was associated with differential activation primarily in temporal, supramarginal, and precentral regions. For social distance, no clusters survived correction for either near > far or far > near. We also tested the fMRI interaction contrasts between objective risk and social distance. No clusters survived correction for either interaction direction.

## 4. Discussion

The present exploratory study examined how objective risk and social distance were associated with trust decisions in a 2 × 2 trust game paradigm. Behaviorally, objective risk showed a clear effect: trust rates were lower under high-risk than low-risk conditions, whereas neither the main effect of social distance nor the interaction reached significance. At the neural level, task-related and decision-related contrasts involved frontal, parietal, temporal, occipital, and insular regions, and the high-risk versus low-risk contrast was associated with differential activation primarily in the supramarginal gyrus (SMG), superior temporal gyrus (STG), middle temporal gyrus (MTG), and precentral gyrus. Under the present task parameters, we did not observe significant whole-brain modulation by social distance. Taken together, these findings suggest that, in this low-context paradigm, objective risk was a more robust source of behavioral variation and whole-brain activation differences than social distance.

The task > baseline and decision > baseline contrasts indicate that trust game performance recruited a distributed set of frontal, parietal, temporal, occipital, and insular regions rather than a single narrowly defined system. Relative to baseline, the task engaged the AG, SMG, superior frontal gyrus (SFG), inferior frontal gyrus (IFG), middle frontal gyrus (MFG), middle occipital gyrus (MOG), IPL, and precentral gyrus, while negative clusters were observed in the insular cortex, STG, postcentral gyrus, and precuneus. During the decision epoch, positive activation was observed primarily in the precentral gyrus, IPL, MFG, superior frontal gyrus, orbital part, middle frontal gyrus, orbital part, and cerebellar vermis. In broad terms, this pattern is consistent with prior accounts that trust under uncertainty recruits systems involved in evaluation, cognitive control, and the integration of social and probabilistic information, especially in frontal and parietal–temporal regions (Miller & Cohen, 2001; Bressler & Menon, 2010; Chen et al., 2020; Krueger & Meyer-Lindenberg, 2019). At the same time, because the present analyses were exploratory and whole-brain in nature, these findings are better interpreted as a descriptive pattern of task engagement than as evidence for a sharply defined network-level mechanism.

The trust > distrust contrast further suggests that choosing to trust was accompanied by greater activation in bilateral IFG and MFG, medial superior frontal and cingulate regions, and visual regions, including the superior occipital gyrus, fusiform gyrus, calcarine sulcus, and lingual gyrus, whereas negative clusters were observed in the STG, insula, and SMG. A cautious interpretation is that trust decisions in the present task were associated with stronger engagement of frontal and visual regions, while distrust-related processing may have placed relatively greater weight on temporal, parietal, and insular regions. This reading is broadly compatible with previous discussions of trust as involving valuation, control, and social appraisal, although the prior literature also suggests that these components are closely intertwined rather than cleanly separable (Miller & Cohen, 2001; Bressler & Menon, 2010; Chen et al., 2020; X. Hu et al., 2018; Krueger & Meyer-Lindenberg, 2019). Earlier work has also linked trusting versus withholding trust to differences in socially evaluative and vigilance-related processing (Van Overwalle, 2009; Brandi et al., 2021), but the present data do not warrant strong claims about a specific network-level dissociation.

The objective-risk contrast was one of the clearest findings in this study. Relative to low risk, high risk was associated with differential activation in the SMG, STG, MTG, and precentral gyrus. This pattern is broadly consistent with the idea that increasing explicit risk changes how participants process the decision context, particularly in temporal and supramarginal regions that may support the integration of social and probabilistic information under uncertainty (Bressler & Menon, 2010; Chen et al., 2020; X. Hu et al., 2018; Krueger & Meyer-Lindenberg, 2019). At the behavioral level, the same manipulation reliably reduced trust choices, in line with earlier work showing that higher objective risk or betrayal-related uncertainty reduces willingness to trust (Bohnet & Zeckhauser, 2004; Yang et al., 2015). More generally, this finding also fits with broader theories of risk perception and uncertainty evaluation, in which explicit loss probability can substantially shape behavior and appraisal (Knight, 1921; Slovic, 1987; Loewenstein et al., 2001). However, this result should still be interpreted with caution. In the present task, objective risk directly altered the payoff structure associated with choosing Trust, whereas choosing Not Trust avoided that added uncertainty. Accordingly, the observed risk effect may reflect a combination of trust-related evaluation and more general expected-value, loss-probability, or risk-aversion processes, rather than a process-pure modulation of social trust alone (Berg et al., 1995; Bohnet & Zeckhauser, 2004; Chetty et al., 2020; Levy & Glimcher, 2012). For this reason, we interpret objective risk here as a robust contextual factor associated with trust behavior and brain activation under the present paradigm, rather than as evidence that we isolated a uniquely social mechanism.

By contrast, we did not observe significant behavioral or whole-brain effects of social distance under the present manipulation. This null result should not be interpreted as evidence that social distance is unimportant for trust more generally. A more cautious interpretation is that the affiliation-based manipulation used here may have been too weak or abstract to produce robust effects in a low-context, largely stranger-based paradigm. Near and far partners differed only by university affiliation, the two universities were located in the same city, and the pilot validation was conducted in a separate sample rather than within the scanned participants. Under these conditions, any influence of social distance may have been attenuated by limited manipulation strength, modest statistical power, or restricted variability in perceived closeness. This interpretation remains compatible with prior behavioral work showing that social closeness can increase trust under richer or more consequential relational settings (Binzel & Fehr, 2013; Rosenberger et al., 2020), with theoretical accounts emphasizing how psychological distance shapes interpretation and construal (Trope & Liberman, 2010), and with broader findings on ingroup favoritism in cooperative behavior (Balliet et al., 2014). Related applied work also suggests that distance cues can influence vigilance and judgment in socio-technical settings (Martin et al., 2021). Thus, the present findings indicate only that no reliable social-distance effect was detected under the current task parameters.

Taken together, the current findings are more consistent with the view that objective and subjective determinants of trust do not always exert equally strong effects within a single task context. Some perspectives suggest that social distance and risk should interact, because relational closeness may alter the subjective meaning of potential loss (Bohnet & Zeckhauser, 2004; Loewenstein et al., 2001; Trope & Liberman, 2010), whereas other perspectives leave open a more additive or partially independent contribution of social and nonsocial inputs (Levy & Glimcher, 2012; Bressler & Menon, 2010; Chen et al., 2020). Under the present low-context manipulation, the observable effect of objective risk was stronger than that of social distance. This does not resolve the broader theoretical question, but it does suggest that an explicit probability structure may dominate weak affiliation cues in one-shot trust settings.

Several limitations should be acknowledged. First, the final fMRI sample was modest (N = 20), which limits statistical power and the stability of whole-brain estimates. Second, the task does not fully dissociate trust-specific social computation from more general risk/value processing, because the objective-risk manipulation affected realized return outcomes only after Trust choices, and those outcomes reflected a combination of trustee behavior and die-roll resolution rather than risk probability alone. By contrast, choosing Not Trust avoided that return-stage uncertainty (Chetty et al., 2020; Levy & Glimcher, 2012). Third, the social-distance manipulation was relatively minimal and was not accompanied by an in-scanner manipulation check, which limits interpretation of the null effect (Binzel & Fehr, 2013; Rosenberger et al., 2020; Trope & Liberman, 2010). Fourth, the whole-brain results were obtained using an exploratory thresholding approach and therefore should be interpreted conservatively. Future work would benefit from larger samples, stronger and more personalized social-distance manipulations, clearer reporting of payoff structure and expected-value relations, and analytic approaches that better separate social, motor, and nonsocial risk-related components of trust decisions (Bressler & Menon, 2010; Miller & Cohen, 2001; X. Hu et al., 2018; Krueger & Meyer-Lindenberg, 2019).

Despite these limitations, the present study contributes a cautious empirical observation: within this exploratory trust game fMRI paradigm, objective risk showed a reliable association with both trust behavior and whole-brain activation differences, whereas social distance did not produce detectable effects under the current manipulation. More broadly, the findings suggest that, in low-context trust settings, explicit risk information may exert a stronger influence on observed decisions than relatively weak affiliation-based distance cues. This conclusion should be understood as specific to the present task parameters and as a starting point for more rigorous future work, rather than as a definitive account of the neural basis of trust.

## 5. Conclusions

In this exploratory fMRI study, objective risk, rather than social distance, was more consistently associated with trust behavior and whole-brain activation differences under the present task parameters. Behaviorally, participants trusted less under high than low objective risk, whereas no reliable main effect of social distance or interaction between objective risk and social distance was observed. At the neural level, objective risk was associated with differential activation primarily in temporal, supramarginal, and precentral regions, including the supramarginal gyrus, superior temporal gyrus, middle temporal gyrus, and precentral gyrus. By contrast, no significant whole-brain modulation by social distance was detected. Given the exploratory design, modest sample size, and the task’s limited ability to disentangle social trust from more general risk/value processing, these findings should be interpreted cautiously. Overall, the results suggest that, in low-context trust settings, explicit risk information may exert a stronger influence on observed decisions than relatively weak affiliation-based distance cues.

## Figures and Tables

**Table 1:** Significant clusters in whole-brain analysis for task > baseline.

Brain Region	Left/Right (L/R)	Brodmann Area (BA)	MNI Coordinates (x, y, z)	t-Value	Z-Score	Cluster Size (k)
**Positive Activation**						
Angular Gyrus	R	39	28, −52, 38	7.318	4.987	646
Supramarginal Gyrus	R	48	36, −50, 38	6.362	4.601	
Angular Gyrus	R	39	46, −38, 36	4.103	3.429	
Superior Frontal Gyrus	R	9	14, 48, 32	7.247	4.960	4042
Superior Frontal Gyrus	R	10	20, 58, 26	6.904	4.827	
Medial Superior Frontal Gyrus	R	10	12, 36, 44	6.288	4.569	
Inferior Frontal Gyrus	R	45	44, 30, 30	6.206	4.533	2015
Precentral Gyrus	R	6	44, 8, 30	6.130	4.499	
Middle Frontal Gyrus	R	6/8	44, 36, 24	5.644	4.273	
Middle Occipital Gyrus	L	7	−28, −58, 38	5.972	4.428	888
Inferior Parietal Lobule	L	40	−32, −48, 30	4.771	3.821	
Postcentral Gyrus	L	6	−40, −30, 42	4.593	3.721	
Precentral Gyrus	L	6	−40, 2, 34	5.549	4.227	2220
Inferior Frontal Gyrus, Orbital Part	L	11	−54, 14, 22	4.842	3.860	
Insular Cortex	L	48	−30, 6, 18	4.834	3.856	
Middle Frontal Gyrus	L	47	−36, 58, −4	4.442	3.633	391
Middle Frontal Gyrus	L	10	−38, 60, 8	4.147	3.456	
Middle Frontal Gyrus	L	10	−28, 54, 10	3.900	3.301	
**Negative Activation**						
Insular Cortex	R	48	38, −18, 14	10.797	6.043	8866
Superior Temporal Gyrus	R	42	42, −30, 18	9.299	5.643	
Superior Temporal Gyrus	R	22	50, −24, 8	8.322	5.340	
Postcentral Gyrus	R	3	28, −38, 50	6.703	4.745	1766
Precuneus	L	23	−12, −44, 48	6.374	4.606	

Note: BA—Brodmann area, MNI—Montreal Neurological Institute coordinates, k—cluster size in voxels.

**Table 2:** Significant clusters for decision screen > baseline contrast.

Brain Region	Left/Right (L/R)	Brodmann Area (BA)	MNI Coordinates (x, y, z)	t-Value	Z-Score	Cluster Size (k)
**Positive Activation**						
Precentral Gyrus	L	6	−56, 6, 26	12.646	6.458	30,437
Inferior Parietal Lobule	L	40	−28, −42, 42	12.009	6.324	
Inferior Parietal Lobule	L	41	−32, −52, 40	11.738	6.264	
Middle Frontal Gyrus	L	10	−32, 58, 8	6.327	4.586	595
Superior Frontal Gyrus, Orbital Part	L	11	−22, 68, −2	5.595	4.250	
Middle Frontal Gyrus, Orbital Part	L	10	−36, 60, −4	5.078	3.987	
Cerebellar Vermis Areas 4/5			0, −60, −8	5.609	4.257	322
Cerebellar Vermis Areas 4/5			4, −50, −12	5.449	4.178	
Cerebellar Areas 4/5	R		14, −46, −18	5.216	4.060	

**Table 3:** Significant clusters for the trust > distrust contrast.

Brain Region	Left/Right (L/R)	Brodmann Area (BA)	MNI Coordinates (x, y, z)	t-Value	Z-Score	Cluster Size (k)
**Positive Activation**						
Inferior Frontal Gyrus	R	44	40, 10, 32	6.900	4.825	1233
Inferior Frontal Gyrus	R	48	40, 28, 26	6.489	4.656	
Middle Frontal Gyrus	R	45	44, 32, 20	5.853	4.372	
Precentral Gyrus	L	6	−36, 6, 32	6.242	4.549	851
Inferior Frontal Gyrus	L	44	−34, 14, 30	5.144	4.022	
Inferior Frontal Gyrus	L	48	−38, 12, 14	4.041	3.391	
Medial Superior Frontal Gyrus	R	8	4, 30, 42	5.637	4.270	919
Cingulate Gyrus	R	32	14, 22, 36	4.931	3.909	
Medial Superior Frontal Gyrus	R	10	8, 24, 46	4.846	3.862	
Superior Occipital Gyrus	R	7	28, −60, 38	5.389	4.148	525
Fusiform Gyrus	R	37	28, −70, 22	4.872	3.877	
Superior Occipital Gyrus	R	7	20, −62, 40	4.071	3.409	
Calcarine Sulcus	R	18	20, −78, 10	4.470	3.650	363
Calcarine Sulcus	R	19	10, −78, 10	4.110	3.434	
Lingual Gyrus	R	19	22, −68, 6	3.704	3.174	
Middle Frontal Gyrus	R	10	32, 52, 6	4.369	3.591	335
Superior Frontal Gyrus	R	9	34, 66, 2	4.310	3.555	
Middle Frontal Gyrus	R	10	42, 54, 12	3.655	3.141	
**Negative Activation**						
Superior Temporal Gyrus	R	48	50, −26, 18	5.642	4.272	1753
Superior Temporal Gyrus	R	42	56, −18, 16	5.359	4.132	
Insula	R	48	36, −16, 10	4.975	3.932	
Supramarginal Gyrus	L	40	−46, −18, 18	5.550	4.227	1102
Superior Temporal Gyrus	L	42	−46, −32, 10	4.016	3.375	
Insula	L	48	−44, −6, −2	3.890	3.295	

Note: Negative clusters indicate relatively greater activation for distrust than trust.

**Table 4:** Significant clusters for the high risk > low risk contrast.

Brain Region	Left/Right (L/R)	Brodmann Area (BA)	MNI Coordinates (x, y, z)	t-Value	Z-Score	Cluster Size (k)
**Positive Activation**						
Middle Temporal Gyrus	R	21	44, −48, 14	5.463	4.185	743
Supramarginal Gyrus	R	40	64, −26, 26	4.681	3.771	
Superior Temporal Gyrus	R	42	50, −42, 16	4.430	3.626	
Superior Temporal Gyrus	L	48	−50, −26, 30	5.092	3.995	379
Supramarginal Gyrus	L	48	−54, −22, 24	4.349	3.579	
Superior Temporal Gyrus	L	22	−66, −28, 14	3.666	3.148	
Precentral Gyrus	L	6	−50, 8, 16	4.687	3.774	1083
Precentral Gyrus	L	6	−56, 4, 8	4.528	3.684	
Precentral Gyrus	L	6	−50, 0, 30	4.242	3.514	

## Data Availability

Data and procedures necessary to reproduce the experiment and analyses are available online at https://osf.io/9n28m/ (accessed on 17 February 2025).
